# Carcinoma Lung Presenting with Skeletal Muscle Metastasis: Case Report with Review of Literature

**DOI:** 10.1055/s-0041-1731636

**Published:** 2021-06-23

**Authors:** Ankit Lalchandani, Yogeshwar Shukla, Mohammad Masoom Parwez, Vinay Kumar

**Affiliations:** 1 Department of General Surgery, All India Institute of Medical Sciences, Bhopal, Madhya Pradesh, India; 2 Department of Surgical Oncology, All India Institute of Medical Sciences, Bhopal, Madhya Pradesh, India

**Keywords:** lung cancer, skeletal muscle metastasis, soft tissue metastasis, carcinoma lung

## Abstract

Lung cancers usually present very late with distant metastasis, thereby carrying a poor prognosis. Metastasis at unusual sites such as extremity musculature does create a diagnostic challenge. This leads to delay in diagnosis and treatment initiation and further worsens the prognosis of the patient. Not many cases have been reported as of now and no standard guidelines are available regarding clinical approach in such cases. We have presented one of such cases to emphasize on importance of early detection and differentiation of such lesions from primary soft tissue malignancies.

Lung cancer is one of the commonest cancers and cause of cancer-related deaths worldwide. Histopathologically, they are classified as small cell and nonsmall carcinomas, including variants such as squamous cell carcinoma, adenocarcinoma, and large cell carcinoma. It carries a poor prognosis as patients usually present in stage IV disease. However, skeletal muscle metastasis is infrequent, and this being the only presenting symptom is extremely rare. We present a case of a patient of primary adenocarcinoma of lung presenting with thigh muscle metastasis.

## Case Summary

**Fig. 1:**
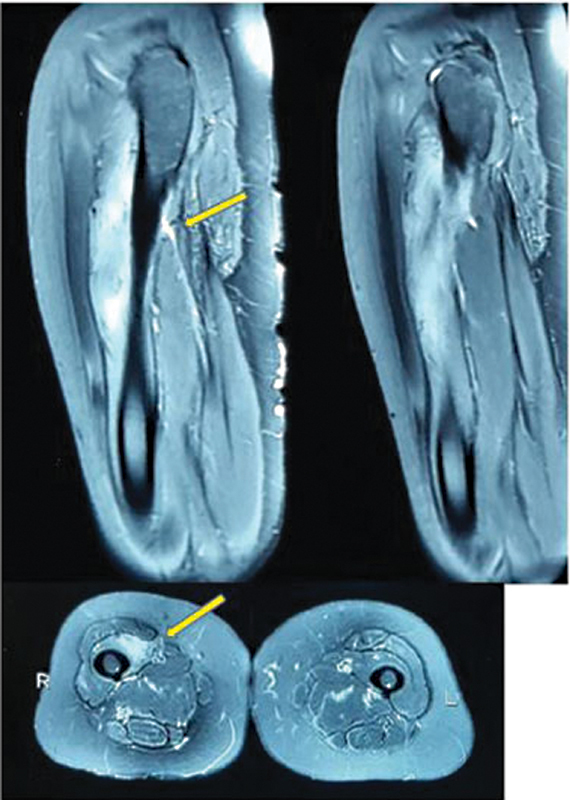
Metastatic lesion in vastus intermedius (yellow arrow).

**Fig. 2:**
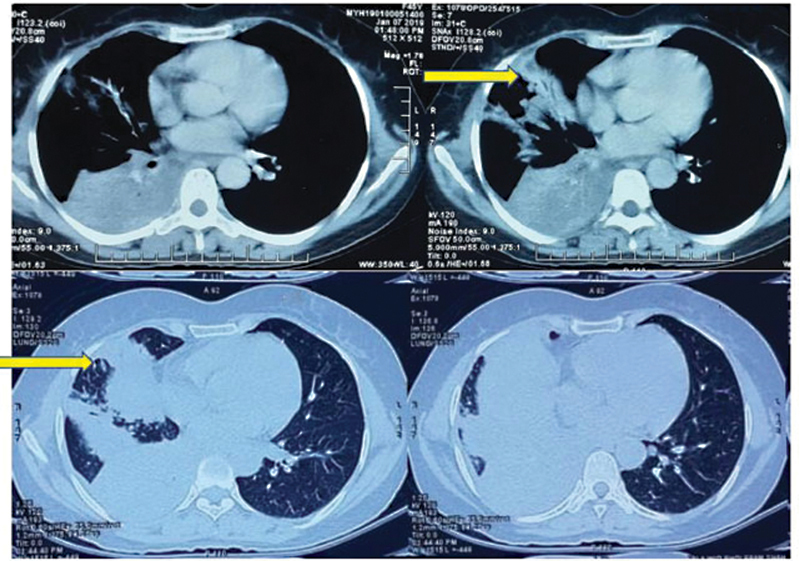
Primary lesion in second lobe of lung (yellow arrow).

A 45-year-old female presented with complaint of pain over the right thigh with difficulty flexing the thigh for 12 months. Pain was nonradiating, and aggravated by brisk walking Patient also complained of loss of appetite and weight but no history of fever, night sweats, or chronic cough. On examination, a vague mass was palpable over the anterior aspect of thigh that appeared to arise from the rectus femoris. There was no sensory or motor deficit. A magnetic resonance imaging (MRI) showed an ill-defined soft tissue mass (3.3 × 3 cm) with few hyperintense areas within, involving vastus intermedius muscle ( Fig. 1 ). An ultrasonography-guided biopsy was taken from the mass that showed metastatic deposits of moderately differentiated adenocarcinoma. On further evaluation for the primary, chest X-ray showed a mass lesion in right middle lobe causing collapse of right lower lobe. High-resolution computed tomography further showed a lesion with homogenous ground glass density with visible pulmonary vasculature suggestive of right lobar consolidation with subcentimetric lymph nodes in the pretracheal and paratracheal region ( Fig. 2 ). Computed tomography-guided biopsy taken from the lesion suggested invasive adenocarcinoma (moderately differentiated). She has been on chemotherapy since.

## Discussion

Patients with lung cancer present in advanced stages with metastasis. They tend to metastasize both via lymphatics and hematogenous route. Metastases are increasingly seen in adenocarcinomas, whereas squamous cell carcinomas have a tendency to locally invade the thoracic wall. 1 Major sites of metastasis are brain, bone, liver, and adrenals. Hematogenous metastasis to skeletal muscle is very rare, even in autopsy studies. Out of 500 autopsies conducted by Willis in carcinoma patients, only four were found to have skeletal muscle metastasis. 2 Even the cancers, which commonly metastasize to the bone such as prostate, breast, and thyroid cancers, only rarely disseminate to soft tissues. 3 According to one of the hypotheses, muscle contractions may prevent metastasis by inducing a high tissue pressure and variable local blood flow, thereby discouraging implantation of tumor cells. 4 According to prior wittich in 1854 provided the first description of a muscle metastasis and Willis was the first to report a muscle metastasis of lung origin. 5 In their case series, Koike et al reported seven cases of carcinoma presenting with skeletal muscle metastases. Four of them were found to have pulmonary origin, of which two were adenocarcinomas and two squamous cell carcinomas. 6

In their review of 118 cases presenting with soft tissue metastasis, Jose Antonio et al identified 13 as having a primary tumor in the lung. Out of them 11 were adenocarcinomas and 2 had squamous cell histology. Upper trunk was the most frequent site for metastasis. 7 Tuoheti et al published a clinicopathological study of 12 patients with skeletal muscle metastasis. Out of which four were of pulmonary origin. About 66% were metastasis to the lower limb. 8 Baldeo et al have reported two patients who presented with lesions over the gluteal region and subscapular region, respectively. Initially referred to general surgeon by their primary physician, they were subsequently found to be have primary lung cancer. 9

Pain is the most frequent symptom (83%), and a mass is palpable in 78% of cases of soft tissue metastases. 10 Metastatic soft tissue masses have high chance of misdiagnosis as the clinical features can mimic a sarcoma. Clinically metastatic masses are painful, while sarcomas are present with painless enlarging masses. MRI and tissue biopsy would help differentiate between the two. Positron emission tomography scan can be of help in identifying any distant primary tumor in case cross-sectional imaging is equivocal.

On MRI, soft tissue metastases are of low or intermediate signal intensity compared with normal muscle tissue on T1-weighted sequences and high signal intensity on T2-weighted sequences, whereas soft tissue sarcomas have a heterogenous T2 signal intensities with peritumoral postcontrast enhancement and the presence of necrotic areas. 11 Poorly differentiated lung carcinoma does not show characteristic histological features, and positive immunohistochemical staining for TTF-1 (Thyroid Transcription Factor), TP53, p40, etc., can be used to clinch the diagnosis. 12 Soft tissue sarcomas, on the other hand, may show spindle cells with epithelioid component and variable staining depending on the subtype. Hence, it is preferable to go with a panel of immunohistochemistry markers at the beginning itself.

We would like to emphasize that it is important to conclusively differentiate between the two as the treatment outcomes and prognosis are different. Soft tissue metastasis in lung cancer has been considered a grave prognostic indicator with a median survival of less than 6 months even on chemotherapy. In a review by Pop et al, among 16 such patients, 5-year survival rate was 11.5% with a median survival duration of 6 months. Prognosis was better in patients with disease-free interval of more than 6 months and a single metastatic lesion, while patients with disease-free interval of less than 6 months and multiple metastasis had 0% survival after 5 years. 10 On the other hand, soft tissue sarcomas of trunk and extremities are amenable to surgery and radiation with a 5 years survival rates being 86, 71, and 51% for stage I, stage II, and stage III, respectively. 13 But due to lack of any large case series on the subject, no definite guidelines on the management of patients with soft tissue metastases of lung origin are currently available. Although the general consensus in the case of metastatic lung cancer is to go for chemotherapy, surgical excision may be indicated for isolated lesions after a long disease-free interval, in tumors with good prognosis or after an appropriate treatment of the primary tumor. 14

Therefore, any soft tissue lesion should be seen with high suspicion of harboring a distant primary tumor until proven otherwise.

## Notes

**Erratum:** Article has been corrected as per Erratum (DOI: 10.1055/s-0043-1769507).
