# Erratum to: Carcinoma Lung Presenting with Skeletal Muscle Metastasis: Case Report with Review of Literature

**DOI:** 10.1055/s-0043-1769507

**Published:** 2023-06-12

**Authors:** Ankit Lalchandani, Yogeshwar Shukla, Mohammad Masoom Parwez, Vinay Kumar

**Affiliations:** 1 Department of General Surgery, All India Institute of Medical Sciences, Bhopal, Madhya Pradesh, India; 2 Department of Surgical Oncology, All India Institute of Medical Sciences, Bhopal, Madhya Pradesh, India

Authors of the above-mentioned article wish to add Dr Vinay Kumar to the author list. The original article was published online in 2021 in issue 07(02) (pp. e121–e123). DOI: 10.1055/s-0041-1731636 .

He contributed in conceptualizing of the manuscript. Author list has been updated as above in the original article.

